# Leptin ameliorates ischemic necrosis of the femoral head in rats with obesity induced by a high-fat diet

**DOI:** 10.1038/srep09397

**Published:** 2015-03-23

**Authors:** Lu Zhou, Kyu Yun Jang, Young Jae Moon, Sajeev Wagle, Kyoung Min Kim, Kwang Bok Lee, Byung-Hyun Park, Jung Ryul Kim

**Affiliations:** 1 Department of Orthopaedic Surgery, Chonbuk National University Medical School, Research Institute for Endocrine Sciences and Research Institute of Clinical Medicine of Chonbuk National University-Biomedical Research Institute of Chonbuk National University Hospital, Jeonju, Republic of Korea; 2 Department of Pathology, Chonbuk National University Medical School, Research Institute for Endocrine Sciences and Research Institute of Clinical Medicine of Chonbuk National University-Biomedical Research Institute of Chonbuk National University Hospital, Jeonju, Republic of Korea; 3 Department of Biochemistry, Chonbuk National University Medical School, Research Institute for Endocrine Sciences, Jeonju, Republic of Korea; 4 Department of Sports Medicine, Taishan Medical University, Shandong, China

## Abstract

Obesity is a risk factor for ischemic necrosis of the femoral head (INFH). The purpose of this study was to determine if leptin treatment of INFH stimulates new bone formation to preserve femoral head shape in rats with diet-induced obesity. Rats were fed a high-fat diet (HFD) or normal chow diet (NCD) for 16 weeks to induce progressive development of obesity. Avascular necrosis of the femoral head (AVN) was surgically induced. Adenovirus-mediated introduction of the leptin gene was by intravenous injection 2 days before surgery-induced AVN. At 6 weeks post-surgery, radiologic and histomorphometric assessments were performed. Leptin signaling in tissues was examined by Western blot. Osteogenic markers were analyzed by real-time RT-PCR. Radiographs showed better preservation of femoral head architecture in the HFD-AVN-Leptin group than the HFD-AVN and HFD-AVN-LacZ groups. Histology and immunohistochemistry revealed the HFD-AVN-Leptin group had significantly increased osteoblastic proliferation and vascularity in infarcted femoral heads compared with the HFD-AVN and HFD-AVN-LacZ groups. Intravenous injection of leptin enhanced serum VEGF levels and activated HIF-1α pathways. Runx 2 and its target genes were significantly upregulated in the HFD-AVN-Leptin group. These results indicate that leptin resistance is important in INFH pathogenesis. Leptin therapy could be a new strategy for INFH.

Legg-Calve-Perthes disease (LCPD) is a common juvenile form of ischemic osteonecrosis of the femoral head (INFH) that can lead to permanent femoral head deformity and end-stage osteoarthritis, even in young adults^1^. INFH remains one of the most challenging conditions to treat because little is understood regarding the biology of the disease. Both vascular disruption and defective bone repair are key elements of INFH pathogenesis^2^. Because joint replacement arthroplasty is unsuitable for young people, biological treatments to preserve the femoral head structure more effectively are needed.

Obesity is a clinical risk factor for LCPD and poor treatment outcomes are associated with high body mass index (BMI)^3^. Leptin, the product of the *ob* gene, is an adipocyte-produced 146 amino acid polypeptide that regulates adipose tissue mass and body weight^4^,^5^. Most obese humans have very high plasma leptin concentrations, suggesting they are resistant to its anorectic and metabolic effects^6^. In obese, leptin-deficient mice (*ob/ob*), administering exogenous leptin effectively reduces hyperphagia and obesity^7^,^8^. Recently, we measured circulating leptin levels, soluble leptin receptor levels, and the free leptin index (FLI) in LCPD patients and healthy controls^3^. In LCPD patients, circulating leptin levels were significantly higher and soluble leptin receptor levels were significantly lower than in controls. In addition, FLI was higher in the LCPD group than in controls.

Recent studies have shown that leptin increased in bone formation in leptin deficient mice^9^,^10^,^11^. To our knowledge, the therapeutic potential of leptin to increase bone formation and angiogenesis after ischemic osteonecrosis has not been studied. The purpose of this study was to determine if systemic leptin administration stimulated bone formation and preserved femoral head shape in INFH in rats with induced obesity.

## Results

Rats returned to normal activity within 24 h after surgery. Five rats died during the postoperative period: one each from the NCD-AVN, HFD-AVN and HFD-AVN-Leptin groups and two from the HFD-AVN-LacZ group. The remaining 115 animals experienced an uncomplicated postoperative course until six weeks post-surgery. For induction of AVN, the femoral heads were dislocated from the acetabulum and then the dislocated femoral heads were re-located to the acetabulum. However, since the rat's acetabulum is extremely shallow, all the hips became re-dislocated, except in the sham group, as evidenced by all the rats walked with a limp on their dislocated hips.

Consumption of a HFD for 16 weeks produced a significant (P < 0.05) increase in body weight compared to the consumption of a NCD. The mean plasma leptin level at the time of necropsy in NCD-Sham, HFD-Sham, NCD-AVN, HFD-AVN, and HFD-AVN-LacZ animals averaged 1.5 ± 0.4, 28.3 ± 1.2, 1.3 ± 0.1, 27.3 ± 2.1, and 26.8 ± 1.6 ng/ml, respectively. In HFD-AVN-leptin rats, the mean plasma leptin level rose within 2 days to 73.5 ± 3.4 ng/ml and remained elevated for 2 weeks after Ad-leptin injection. After the surgery, a reduction in body weight was not observed in NCD-Sham, HFD-Sham, NCD-AVN, HFD-AVN, and HFD-AVN-LacZ groups. In contrast, the HFD rats treated with Ad-Leptin exhibited a 41% reduction in body weight over the 6-weeks period of observation after surgery (Table 1 and Supplementary Fig. 1).

### Micro-CT assessment of ischemic osteonecrosis

**Figure 1:**
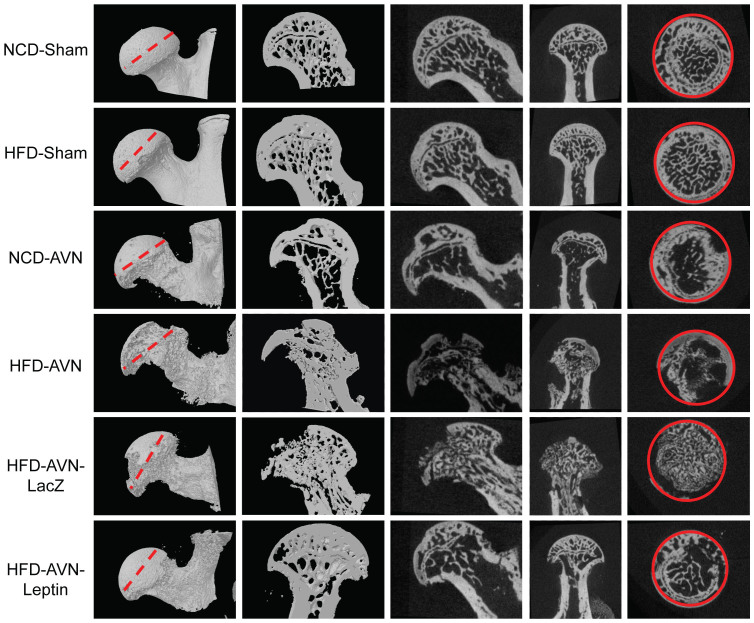
Radiographs and histological findings for infarcted femoral heads. Micro-CT scan images of infarcted femoral heads by groups. Femoral heads showed significantly lower trabecular bone in the HFD-AVN and HFD-AVN-LacZ groups compared with the NCD-AVN group; the trabecular networks were preserved in the HFD-AVN-Leptin group. ROIs (solid red lines) of groups were cross-sectional areas of the middle of femoral heads (dotted red lines).

Structural and quantitative assessment of mineralized skeletal tissue formation was performed by micro-CT at 6 weeks after surgery-induced INFH. Femoral heads in the NCD-sham and HFD-sham group animals did not develop osteonecrosis (Fig. 1). However, femoral heads in HFD-AVN-group animals showed significant loss of trabecular bone and reduced preservation of femoral head architecture compared with the NCD-AVN group. Femoral heads in HFD-AVN-LacZ-group animals also showed a prominent area of bone resorption. In contrast, the trabecular network and femoral head architecture were relatively well preserved in the HFD-AVN-Leptin group compared to the HFD-AVN or HFD-AVN-LacZ group. Micro-CT findings were used to measure bone volume, trabecular numbers, and trabecular thicknesses (Table 2). Bone volume (BV) in the HFD-AVN-Leptin group in areas undergoing repair 6 weeks after surgery was significantly higher than in the HFD-AVN and HFD-AVN-LacZ groups. Mean BV/TV values and trabecular numbers were significantly lower in femoral heads in the HFD-AVN and HFD-AVN-LacZ groups compared to the NCD-AVN group, whereas the HFD-AVN-Leptin group had bone masses and microarchitectures similar to the sham group. In addition, trabecular number and thickness were significantly higher and trabecular separation was significantly lower in the HFD-AVN-Leptin group compared to the HFD-AVN or HFD-AVN-LacZ group.

### Histological assessments

**Figure 2:**
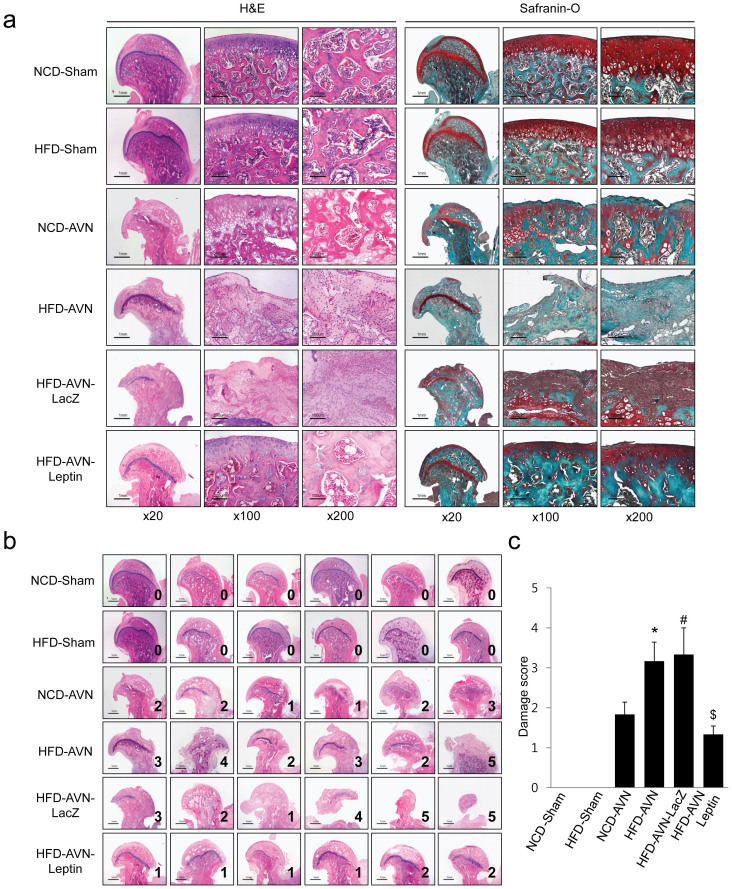
Histologic findings of sham and infarcted femoral heads. (a) Histological results of femoral heads from sham-operated groups (NCD-Sham and HFD-Sham) showing intact, smooth articular surface and damage to articular surfaces in AVN-induced groups (NCD-AVN, HFD-AVN, and HFD-AVN-LacZ). Secondary ossification centers were replaced with fibrovascular tissue. Femoral heads of HFD-AVN and HFD-AVN-LacZ groups showed more deformity compared to the NCD-AVN group. HFD-AVN and HFD-AVN-LacZ groups showed increases in damaged of epiphyseal cartilage surfaces and metaphyseal physes and secondary ossification centers replaced by fibrous tissue. Femoral heads of HFD-AVN-Leptin group were preserved with intact articular cartilage and ossification center marrow. (c) Numbers from (b) are damage scores. *, p < 0.01 vs. NCD-AVN; ^#^, p < 0.01 vs. NCD-AVN; ^$^, p < 0.05 vs. HFD-AVN. Five rats were used in each groups.

Histologically, femoral heads of NCD-sham and HFD-sham animals were intact and articular surface and secondary ossification centers were well preserved. However, femoral heads in the HFD-AVN and HFD-AVN-LacZ groups were damaged. Articular surfaces and metaphyseal physes were also damaged and secondary ossification centers were collapsed and replaced with nonhematopoietic fibrovascular tissue (Fig. 2a). Although femoral heads in the NCD-AVN group were also damaged, the degree of deformity was less than in the HFD-AVN or HFD-AVN-LacZ groups. Damage scores for the HFD-AVN or HFD-AVN-LacZ groups were significantly higher than the NCD-AVN group (p < 0.05) (Fig. 2b, c). In contrast, femoral heads in the HFD-AVN-Leptin group were relatively well preserved compared to those in the HFD-AVN or HFD-AVN-LacZ groups. Although articular surfaces were slightly irregular and showed detachment of the articular cartilage, the general contour of the articular surface was maintained. Secondary ossification centers were intact and hematopoietic. Bone in secondary ossification centers was rimmed with osteoblasts and well-formed blood vessels in the bone marrow (Fig. 2a). Damage scores were significantly lower in the HFD-AVN-Leptin group compared with the HFD-AVN or HFD-AVN-LacZ groups (p < 0.01) (Fig. 2b, c).

### Enhanced vascularity induced by leptin

**Figure 3:**
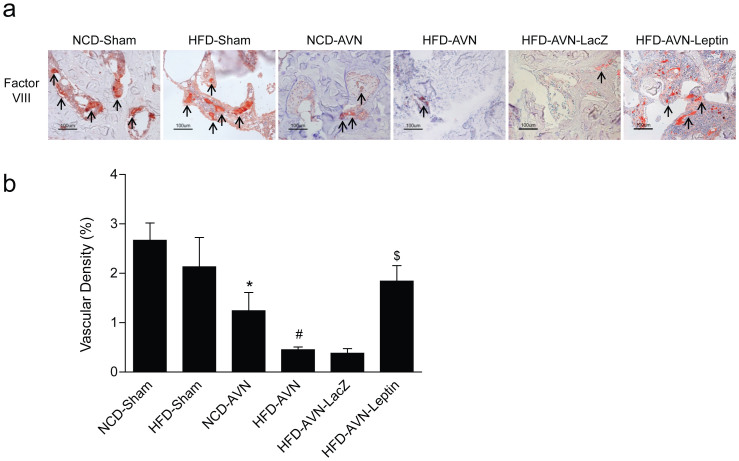
Immunohistochemical staining for OB-R and factor VIII-related antigen and vascular densities in femoral heads. (a) Immunohistochemical staining for factor VIII-related antigen. Fewer vessels were positive for factor VIII-related antigen in NCD-AVN and HFD-AVN groups than NCD-Sham or HFD-Sham groups. HFD-AVN animals had fewer blood vessels. HFD-AVN-Leptin had a high number of vessels positive for factor VIII-related antigen. Arrows, factor VIII-related antigen-positive blood vessels. (b) Femoral head vascular densities by group. Vascular density (%) was calculated as vascular area stained by antibody for factor VIII-related antigen/total area of each image x 100. Data are mean ± SEM (n = 6 per group). *, p < 0.01 vs. NCD-Sham; ^#^, p < 0.01 vs. NCD-AVN; ^$^, p < 0.01 vs. HFD-AVN-LacZ.

To evaluate the effects of leptin on femoral head vascularity, femoral heads were immunostained for factor VIII-related antigen. Immunostaining for factor VIII-related antigen revealed more blood vessels in sham-operated and the HFD-AVN-Leptin groups compared with the HFD-AVN or HFD-AVN-LacZ groups (Fig. 3a). Elevated vessel density in the HFD-AVN-Leptin group was confirmed by morphometric analysis for vessels in the secondary ossification centers of infarcted femoral heads. Vascular densities in the HFD-AVN and HFD-AVN-LacZ groups were significantly lower than in the sham-operated groups (p < 0.01). However, vascular density of the HFD-AVN-Leptin group was significantly higher than in the HFD-AVN or HFD-AVN-LacZ groups (p < 0.01) (Fig. 3b). Vascularization of the femoral head represented by vascular density paralleled radiological and histological findings.

### Leptin induces increases in VEGF

**Figure 4:**
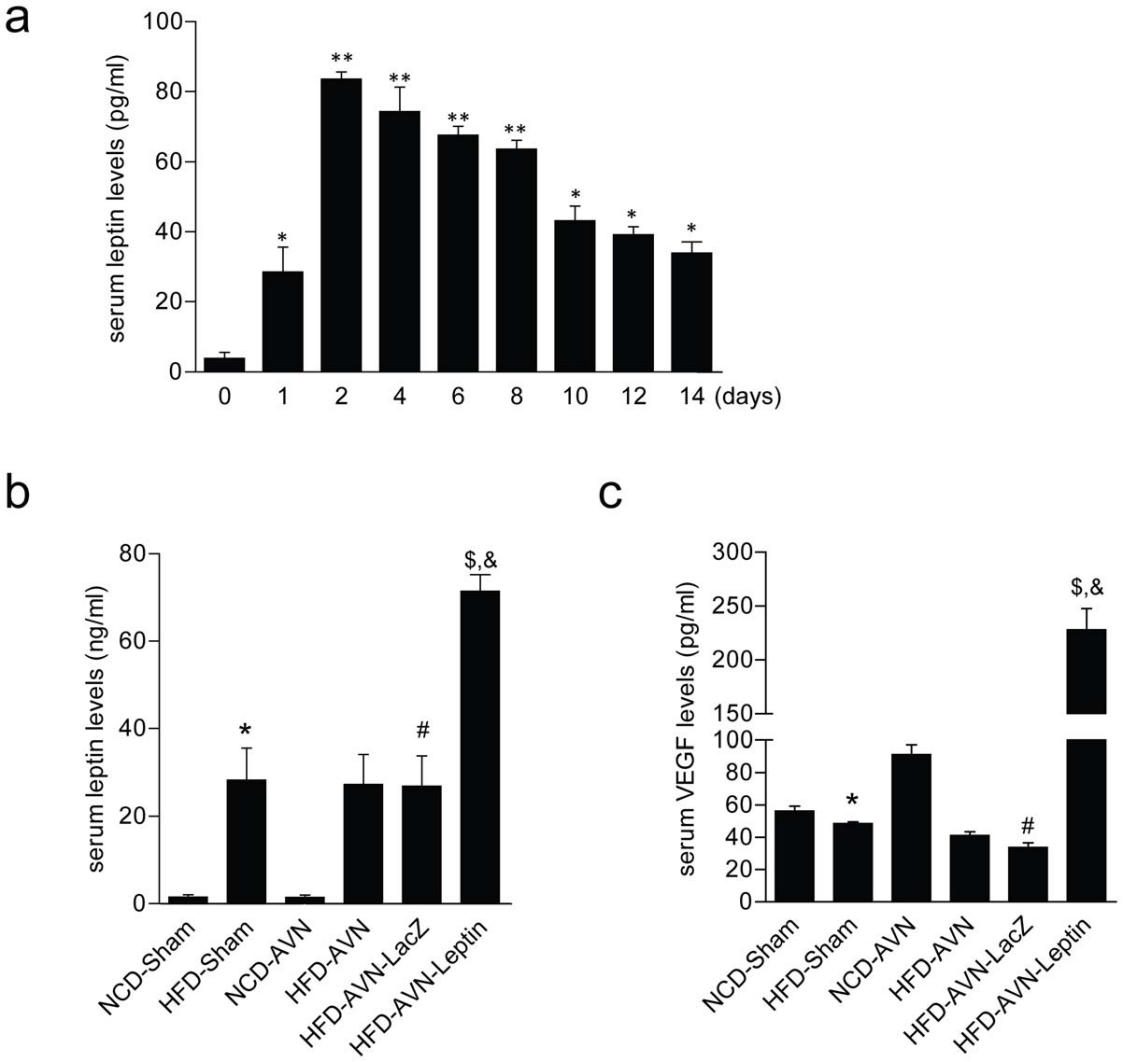
Time-dependent expression of leptin and serum levels of leptin and VEGF by group. (a) Leptin adenovirus was injected two days before surgery and blood was extracted every two days and analyzed by ELISA. (B and C) One week after operation, blood was extracted from tail veins and analyzed for leptin (b) and VEGF (c) by ELISA. Shown: leptin treatment group with high leptin and VEGF. Data are expressed as the mean SEM (n = 4 per group). *p < 0.05 vs day 0; ** *p* < 0.01 vs. day 0. *, p < 0.05 vs. NCD-Sham; ^#^, p < 0.05 vs. NCD-AVN; ^$^, p < 0.01 vs. NCD-AVN; ^&^, p < 0.01 vs. HFD-AVN-LacZ.

Serum leptin levels peaked two days after Ad-Leptin injection and slowly declined over 10 days, maintaining a concentration of approximately 40 ng/mL until 14 days (Fig. 4a). In experimental groups, serum leptin levels were significantly higher in the HFD-AVN-Leptin group compared to the HFD-AVN or HFD-AVN-LacZ groups (Fig. 4b). Serum VEGF levels were consistently higher with increasing serum leptin after Ad-Leptin treatment in the HFD-AVN-Leptin group compared to the HFD-AVN or HFD-AVN-LacZ groups (Fig. 4c).

### Leptin expression associates with HIF1α -related pathways

**Figure 5:**
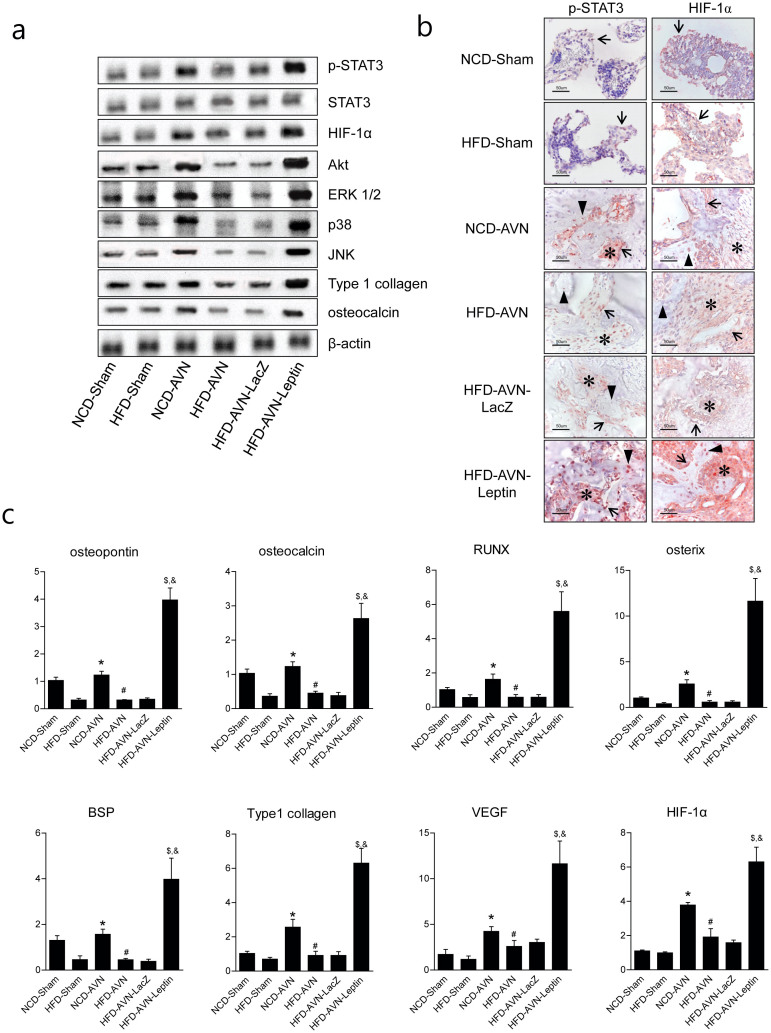
Leptin increased VEGF levels via canonic and noncanonical signaling pathways. (a) Western blot analysis of p-STAT3, HIF-1a, ERK1/2, Akt, p38, osteocalcin and collagen I in infracted femoral heads by group. (b) Expression of phosphorylated STAT3 and HIF-1α were stronger in osteoblasts (arrows), osteoblasts (arrow heads), and stromal fibroblasts (asterisks) in HFD-AVN-Leptin compared with HFD-AVN or HFD-AVN-LacZ. Phosphorylated STAT3 and HIF-1α expression in NCD-AVN was intermediate between HFD-AVN-Leptin and HFD-AVN. (c) Quantification analysis of mRNA of HIF-1α, VEGF and related osteogenic genes in femoral heads by real-time RT-PCR by group. PCR reactions were run in triplicates and performed twice. *, p < 0.05 vs. NCD-Sham group; ^#^, p < 0.05 vs. NCD-AVN group; ^$^, p < 0.01 vs. NCD-AVN group; ^&^, p < 0.01 vs. HFD-AVN-LacZ group. Although cropped blots were used, the gels were run under the same experimental conditions.

Western blots were used to determine the impact of leptin treatment. Leptin treatment was associated with increase in STAT3 phosphorylation, increase expression of the leptin canonic signaling kinases MAPK/ERK 1/2 and PI-3K/AKT1 and noncanonical JNK and P38. In addition, HIF-1α expression also increased in the HFD-AVN-Leptin group compared to the HFD-AVN and HFD-AVN-LacZ groups (Fig. 5a, Supplementary Fig. 2) and the immunohistochemical expression of p-STAT3 and HIF-1α was similar to the results of western blots (Fig. 5b). Expression of p-STAT3 and HIF-1α were seen in stromal cells on bone surfaces (which are likely either osteoblasts or bone lining cells) and was stronger in the HFD-AVN-Leptin group than the HFD-AVN or HFD-AVN-LacZ groups (Supplementary Fig. 3). Expression of phosphorylated STAT3 and HIF-1α in the NCD-AVN group was intermediate between its expression levels in the HFD-AVN-Leptin and HFD-AVN groups. Real-time RT-PCR was performed to determine the mRNA levels for HIF-1α and VEGF. Leptin treatment increased HIF-1α and VEGF mRNA in the HFD-AVN-Leptin group compared to the HFD-AVN and HFD-AVN-LacZ groups (Figure 5c). These results suggest that Ad-Leptin treatment causes an increase in angiogenesis and that might be related to the canonical (MAPK and PI-3K) and noncanonical (JNK and p38 kinase) leptin signaling pathways in INFH.

### The effect of leptin for the genes related to osteogenesis

mRNA was isolated from necrosis areas of the femoral heads and assayed for the expression of several osteogenic genes: runt-related transcription factor 2 (Runx2), bone sialoprotein, type 1 collagen, osteopontin, and osterix. Expressions of the osteogenic genes were significantly lower in the HFD-sham groups compared to the NCD-sham group. The NCD-AVN group showed higher levels of osteogenic genes compared to NCD-sham group, whereas, there were no differences between the HFD-sham group and the HFD-AVN group. Expressions of the osteogenic genes were significantly lower in the HFD-AVN and HFD-AVN-LacZ groups compared to the HFD-AVN-Leptin group. The HFD-AVN-Leptin group showed a higher level of expression of osteogenic genes than the other groups (Fig. 5c).

## Discussion

In this study, we found that INFH in rats with diet-induced obesity was strongly associated with severe destruction of bone architecture and reduced potential for bone regeneration. We examined the effects of leptin on revascularization and repair of the femoral head in rats with diet-induced obesity and INFH. Intravenously injected Ad-leptin effectively facilitated repair of the ischemic femoral head and enhanced angiogenesis and bone regeneration in rats with induced obesity. This study showed that leptin might be useful for the treatment of INFH in obese patient and indicated factors that might predict poor treatment outcome.

The role of leptin in the pathogenesis of obesity can be inferred by measuring plasma leptin^12^. The increase in leptin is a result of obesity as adipocytes secrete leptin. In general, obese animals have higher leptin levels than controls, indicating that these forms of animal obesity are associated with leptin resistance^13^. In our previous study, circulating leptin levels in LCPD patients were significantly higher and levels of soluble leptin receptor were significantly lower than in controls. In addition, the FLI was higher in the LCPD group. Furthermore, the circulating leptin and soluble leptin receptor levels and FLI correlated with LCPD disease severity and treatment outcomes^3^. Therefore, leptin resistance could be relevant to pathogenesis in LCPD, the childhood form of INFH. We suggest that leptin resistance in obesity deteriorates the bone repair process in INFH. In present study, AVN of HFD animals was associated with poor outcome that appear to be improved by intravenous Ad-leptin injection.

Leptin contributes to the formation of a relationship between fat mass and bone^14^ and circulating leptin concentration is related to bone mass. Although Ducy et al. reported that leptin inhibits bone formation *in viv*^15^, other studies found that leptin promotes differentiation into an osteoblast phenotype and increases synthesis of bone matrix proteins such as type I collagen and osteocalcin^16^,^17^. Steppan *et al*. reported that *in vivo* administration of leptin to *ob*/*ob* mice results in increased bone size and mass^18^. The skeletal abnormalities caused by leptin deficiency are markedly attenuated in morbidly obese *ob/ob* mice^14^. Insulin might be involved in the effect of leptin on bone mass since central administration of leptin dramatically decreases circulating insulin concentration^19^,^20^,^21^,^22^. Systemic administration of leptin is associated with reduced insulin concentration^20^,^23^,^24^,^25^, so the positive skeletal effects found in our *in vivo* study emphasize the direct effects of leptin on bone. Both intracerebroventricular and subcutaneous leptin treatment of *ob/ob* mice stimulated bone growth^10^. Recent study reported that peripheral leptin is essential for normal bone resorption and enhances bone formation^9^. Hamrick *et al*. reported peripheral delivery of leptin for 2 weeks increased bone formation in ob/ob mice^26^. We have also shown that peripherally administered leptin in HFD rats resulted in increased osteogenesis and preserved infarcted femoral head although our adenoviral delivery system provides transient leptin exposure during the first two weeks following induction of AVN. However, for clinical application of leptin therapy, a continuous delivery system should be developed because of the long duration of disease of INFH.

Angiogenesis is an early and essential component of the bone repair process^27^. Leptin, initially identified as a pro-angiogenic factor, is also a positive VEGF regulator^28^. Leptin can directly interact with the OB-R receptor in endothelial cells and activate the STAT3 pathway to enhance its DNA binding activity^28^. Leptin also acts as a modulator of other angiogenic factors and has indirect effects on vascular permeability^29^,^30^,^31^. Kim et al. reported that upregulated VEGF in the epiphyseal cartilage in INFH might stimulate vascular invasion into the necrotic area of the epiphyseal cartilage and restore the femoral head^32^. Our results suggest that leptin might induce angiogenesis by enhancing VEGF expression. Furthermore, capillary densities were greater at sites of new bone formation, demonstrating the importance of angiogenesis during new bone formation after administration of Ad-leptin in the HFD-AVN group. The positive relationship between angiogenesis and new bone formation was consistent with results by Ma *et al*., who showed that VEGF upregulation induces new bone formation and promotes the repair process^33^. The mechanisms involved in VEGF upregulation involved both canonical and noncanonical signaling pathways. Normally, leptin increases VEGF by MAPK/ERK 1/2 and PI-3K/AKT1 and it activates a series of transcription factors such as HIF-1α^34^,^35^. INFH is a well-known hypoxic condition implicated in the transcriptional upregulation of VEG^32^. The VEGF promoter contains distal enhancer sites that bind HIF-1α. Leptin induces canonic and noncanonic signaling pathways to activate HIF-1α in INFH. Therefore, we suggest that increased angiogenesis after leptin treatment enhances bone regeneration in ischemic femoral heads.

Although leptin treatment in the HFD-AVN group increased the mRNA osteogenic genes in our study, we could not determine if leptin directly stimulated osteoblastic differentiation. However, our findings were consistent with previous results that leptin enhances osteoblast differentiation and proliferation^16^. Although we did not conclusively demonstrate direct action of leptin, our results suggest that leptin acts on bone by influencing osteoblasts and osteogenic growth factors and newly formed blood vessels that supply oxygen. Turner *et al*. provided strong evidence that leptin acts primarily through peripheral pathways to enhance bone growth and maturation by increasing osteoblast number and activity, and osteoclast activity^9^.

A limitation of this study was that we did not include a leptin treated sham and AVN treated group. Evaluation of leptin treatment in the absence of obesity provides clinically relevant data due to the lack of leptin resistance as a confounder. Another limitation of this study is the occurrence of dislocation after surgery, which could cause changes in bone loading; nevertheless all rats walked with a limp on the dislocated hips. Another limitation of this study was that we did not investigate leptin signaling in the hypothalamus and in sympathetic regulation of bone formation. Further studies on sympathetic regulation in INFH are needed to investigate the specific mechanism of leptin regulation. In summary, the results of this study demonstrated that systemic administration of leptin enhanced bone regeneration and preserved osteonecrotic femoral heads in rats with INFH and induced obesity through increasing vascularity and bone formation. These results suggested that the administration of leptin could be a new therapeutic strategy to improve INFH treatment outcomes.

## Methods

### Animals and surgical procedures

Male Sprague-Dawley rats (eight weeks old, 170–190 g) were used. Rats had free access to water and standard rat chow pellets and were housed under controlled temperature (22 ± 1°C) and humidity (50% to 60%) with a 12-hour light-dark cycle from 7 AM to 7 PM. After acclimatization for 1 week, animals were fed either a NCD or HFD. NCD (by weight) was 21% protein, 4.5% fat, and 52% carbohydrate (3.94 Kcal/g). HFD was 26.5% protein, 1% cholesterol, 0.4% sodium cholate, 35.4% saturated fat (lard), and 26.6% carbohydrate (5.44 Kcal/g). After 16 weeks of NCD or HFD feeding, AVN was induced by surgical application of a tight ligature around the femoral neck, as previously described^2^. Adenovirus-mediated introduction of the leptin gene (Ad-Leptin) was by intravenous injection 2 days before surgery-induced AVN (Supplementary Fig. 4). Animals were divided into six groups: 1) sham-operated, fed NCD (NCD-sham); 2) sham operated, fed HFD (HFD-sham); 3) AVN, fed NCD (NCD-AVN); 4) AVN, fed HFD (HFD-AVN); 5) Ad-LacZ-injected AVN, fed HFD (HFD-AVN-LacZ); and 6) Ad-Leptin-injected AVN, fed HFD (HFD-AVN-Leptin) (n = 20/group). To minimize the unnecessary use of animals two groups that were Ad-LacZ-injected AVN fed NCD and Ad-Leptin-injected AVN fed NCD, were not used in this study as these animals do not develope leptin resistance.

Animals were euthanized by exsanguination under sodium pentobarbital anesthesia at 6 weeks after inducing INFH. Animals were cared for in accordance with the National Institutes of Health Guidelines for Animal Care. All experimental procedures were approved by the Institutional Animal Care and Use Committee at Chonbuk National University (Approval number: CUH 2012-12-008).

### Intravenous administration procedures and groups

At 2 days before surgery, rats were given 100 μL recombinant adenoviruses (1 × 10^12^ plaque-forming units) by intravenous injection to the tail vein. Adenoviruses contained either leptin cDNA (Ad-Leptin) or, as an inactive control, β-galactosidase cDNA (Ad-LacZ) under control of the cytomegalovirus promoter^36^. Ad-Leptin and Ad-LacZ were generously provided by R.H. Unger (University of Texas Southwestern Medical Center, Dallas, TX, USA).

### Assessment of bone destruction by MicroCT

A SKYSCAN 1076 Micro-CT unit (Skyscan, Kontich, Belgium) was used to assess bone volume within defect sites. The X-ray source was set at 75 kV and 100 μA, with a pixel size of 8.8 μm and 400 projections were acquired over an angular range of 180° (angular step of 0.45°). The area included in CT scans was from the upper margin of the femoral head epiphysis to the femoral neck. A global thresholding algorithm was applied at a constant threshold for all specimens. The threshold was the intensity (gray value) that corresponded to ~45% of the average intensity of the intact cortical bone in specimens. Voxels with intensities exceeding the threshold were considered to contain mineralized tissue. All system aspects were operated using Dataviewer software (SkyScan). On stacked, reconstructed micro-CT cross-section images, manual regions of interest (ROIs) of irregular anatomical contour were drawn on transverse images at the middle of the femoral head (Figure 1). ROIs excluded cortical bone. The volume of interest (VOI) was a stack of ROIs drawn over 52 cross-sections, resulting in a height of 0.45 mm. Tb.Th was the trabeculae mean thickness, Tb.Sp was the mean distance between trabeculae and Tb.N was the average number of trabeculae present per unit length. Tb.Th and Tb.Sp were assessed using direct 3D methods and Tb.N was calculated using the formula Tb.N = (BV/TV)/Tb.Th.

### Histological methods

Resected femurs were fixed in 10% neutral buffered formalin and decalcified in 10% EDTA for 10 days or in rapid decalcifying solution (Calci-Clear Rapid, National Diagnostics, Atlanta, GA, USA) for 12 h. Tissue sections were from the femoral head midline as the most representative area. To evaluate histologic findings, paraffin-embedded tissue sections were stained with hematoxylin and eosin or Safranin-O staining (Sigma-Aldrich, MA, USA). Femoral head damage was graded from 0 to 5 as: 0, no damage; 1, mild damage with maintaining femoral head articular cartilage and architecture; 2, destruction of the articular cartilage and collapse of the secondary ossification center but with less than one-third of the femoral head showing destruction; 3; destruction of between one-third and two-thirds of the femoral head; 4, destruction of more than two-thirds of the femoral head; and 5, complete or near-complete femoral head destruction.

### Immunohistochemical staining and vascular density measurements

Tissue sections were deparaffinized. Antigen retrieval was in a pressure cooker with sodium citrate buffer for 12 min. Tissue sections were incubated with antibody for factor VIII-related antigen (1:50, Chemicon, Temecula, CA, USA), Ob-R (1:200, Santa Cruz Biotech, Santa Cruz, CA, USA), phospho-STAT3 (1:200, Cell Signaling, Beverly, MA, USA), or HIF-1α (1:200, Santa Cruz Biotech) overnight at 4°C. For negative controls, sections were incubated in normal mouse IgG (1:100, Santa Cruz Biotech) instead of specific primary antibody. Peroxidase activity was detected using the enzyme substrate 3-amino-9-ethyl carbazole and counterstained with hematoxylin.

Vascular density was evaluated by measuring factor VIII-related antigen-stained areas in ×200 magnification images obtained using a Nikon ECLIPSE E600 microscope with a 20× objective lens (Plan Fluor20/0.50NA, Nikon) and a digital camera (Nikon DXm1200F). Each image was 0.33 mm^2^. Factor VIII-related antigen-stained images were acquired at the most vascularized area of the femoral head from each rat without knowledge of the experimental group. Six images were taken from each of the six rats per group. Vascular densities were measured using an image analysis system (analySIS, Soft Imaging Systems, Germany) and calculated as (factor VIII-stained vascular area/total image area in ×200 magnification) × 100 (%).

### RNA preparation

A subset of rats was euthanized four weeks after surgery. Five rats from each group were evaluated. Total RNA was prepared from the entire mass of regenerated bone using TRIzol reagent (Invitrogen, Carlsbad, CA, USA). Tissue harvesting was performed with a distractor for precision and accuracy. All extraneous soft tissue was cleaned and tissues were snap frozen in liquid nitrogen.

### Real time RT-PCR

RNA from extracted tissues was precipitated with isopropanol and dissolved in DEPC-treated distilled water. Total RNA (500 ng) was treated with RNase-free DNase (Invitrogen) and first-strand cDNA was generated using random hexamer primers from first-strand cDNA synthesis kits according the manufacturer's protocol (Applied Biosystems, Foster City, CA, USA). Specific primers were designed (Table 3) using Primer Express software (Applied Biosystems). GAPDH sequence was used as an invariant control. Real-time RT-PCR reaction mixtures were 10 ng reverse-transcribed total RNA, 2 nM forward and reverse primers, and 5 × PCR master mixture in 10 μL. Reactions were performed in 384-well plates using the ABI Prism 7900 HT Sequence Detection System (Applied Biosystems). Samples from 5 rats were analyzed from each group and RNA from individual rats was analyzed separately.

### Enzyme-linked immunosorbent assay (ELISA)

Approximately 600 μL blood was collected from tail veins into heparinized capillary tubes at indicated times for 5 rats per group. Serum was obtained by centrifugation and stored at −80°C until measurement by enzyme-linked immunosorbent assay (ELISA). Leptin and VEGF levels were determined using Quantikine Kits (R&D Systems, Minneapolis, MN, USA) according to the manufacturer's instructions. Samples were assayed in duplicate.

### Western blot analysis

Whole femoral heads were harvested from five rats per group and decalcified in rapid decalcifying solution (Calci-Clear Rapid) for 12 h. Soft tissue was trimmed from specimens, which was washed with phosphate buffered saline and ground to a fine powder using a manual biopulverizer cooled in liquid nitrogen. Total cell extracts were generated using lysis buffer (Cell Signaling) and centrifugation at 12,000 × *g* for 15 min at 4°C. Quantification of total protein was performed with BCA protein assay reagent (Bio-Rad Laboratories, Hercules, CA, USA). Proteins were resolved on 10% SDS-PAGE gels and transferred to PVDF membranes. After blocking in 5% milk in Tris-buffered saline with 0.1% Tween-20 (TBST), membranes were incubated with primary antibodies for phospho-STAT3, total STAT3, ERK, p38, Akt (Cell Signaling), Osteocalcin (Santa Cruz Biotech), Collagen I (Abcam, Kendall Square, MA, USA) or HIF-1α (Santa Cruz Biotech). After washing, blots were incubated with secondary antibody diluted 1:5000 in TBS-T at room temperature for 1 h. Signals were detected by an enhanced chemiluminescence reagent (Santa Cruz Biotech), according to the manufacturer's instructions. Densitometric analysis was conducted directly on the membrane using an LAS-3000 luminoimage analyzer system (Fujifilm, Tokyo, Japan). Relative phosphorylation of proteins was calculated as the ratio of phosphorylated protein to total protein.

### Statistical analysis

Statistical analyses were by one-way ANOVA followed by post-hoc test. Total bone volumes were compared across groups using a Kruskal-Wallis test (ANOVA by ranks). Data are expressed as mean ± SEM. Differences with p values < 0.05 were considered statistically significant.

## Author Contributions

Conceived and designed the experiments: K.Y.J., B.H.P. and J.R.K. Performed the experiments: L.Z., K.Y.J.,Y.J.M., S.W., K.M.K., K.B.L. and J.R.K. Analyzed the data: L.Z., K.M.K. and J.R.K. Wrote the paper: Lu Zhou, K.Y.J. and J.R.K. J.R.K. takes responsibility for the integrity of the data analysis.

## Supplementary Material

Supplementary Information supplementary information

## Figures and Tables

**Table 1:** Body weight, BMI, serum insulin and leptin levels at the time of necropsy

	NCD-Sham	HFD-Sham	NCD-AVN	HFD-AVN	HFD-AVN-LacZ	HFD-AVN-Leptin
Initial body weight (g)	184.8 ± 4.3	180.1 ± 4.7	180.2 ± 2	184.1 ± 1.1	181.8 ± 1.7	180.2 ± 4.1
Final body weight (g)	276.4 ± 6.8	496.6 ± 11.5^a^	253.4 ± 9.6	514.8 ± 11.1	519.2 ± 7.4	308.5 ± 14.6b,c
BMI (g/cm2)	5.9 ± 0.2	7.2 ± 0.8^a^	5.20 ± 0.57	7.2 ± 0.3	7.2 ± 2.6	5.4 ± 0.6b,c
Leptin (ng/ml)	1.5 ± 0.4	28.3 ± 1.2^a^	1.3 ± 0.1	27.3 ± 2.1	26.8 ± 1. 6	28.4 ± 4.5b,c

All measurements are mean ± SEM.^a^ p < 0.01 vs. NCD-Sham;^b^ p < 0.01 vs. HFD-AVN;^c^ p < 0.05 vs. HFD-AVN-LacZ. Values represent mean ± SEM for five animals in each group.

**Table 2:** Effect of obesity and leptin on ischemic osteonecrosis in femoral heads as assessed by Micro-CT

	NCD-Sham	HFD-Sham	NCD-AVN	HFD-AVN	HFD-AVN-LacZ	HFD-AVN-Leptin
BV (mm^3^)	13.37 ± 3.25	14.47 ± 2.14	8.21 ± 1.98^a^	4.91 ± 1.02^b^	4.78 ± 1.72	10.21 ± 4.02c,d
BV/TV (%)	48.08 ± 6.48	49.48 ± 3.13	39.84 ± 7.60^a^	32.48 ± 3.13^b^	28.18 ± 7.40	47.95 ± 5.67c,d
Tb.Th (mm)	0.24 ± 0.05	0.25 ± 0.05	0.20 ± 0.06^a^	0.18 ± 0.03^b^	0.17 ± 0.07	0.24 ± 0.06c,d
Tb.N (mm^−1^)	2.79 ± 0.32	3.31 ± 0.34	2.28 ± 0.51^a^	1.92 ± 0.94^b^	1.87 ± 0.58	2.42 ± 0. 45c,d
Tb.Sp (mm)	0.22 ± 0.08	0.23 ± 0.05	0.26 ± 0.06^a^	0.31 ± 0.07^b^	0.32 ± 0.07	0.24 ± 0.08c,d

Values represent mean ± SEM for five animals in each group. BV, bone volume; TV, tissue volume; Tb.N, trabecular number; Tb.Sp, trabecular separation; Tb.Th, trabecular thickness.

^a^p < 0.01 vs. NCD-Sham;^b^ p < 0.01 vs. HFD-Sham;^c^ p < 0.05 vs. NCD-AVN;^d^ p < 0.01 vs. HFD-AVN-LacZ.

**Table 3:** Sequences and accession numbers for forward (FOR) and reverse (REV) primers used in real-time RT-PCR

Gene	Sequences for primers	Accession No.
Runx2	FOR: TCCCCGGGAACCAAGAAG	**NM_053470.1**
	REV: GGTCAGAGAACAAACTAGGTTTAGA	
BSP	FOR: CCGGCCACGCTACTTTCTT	**J04215.1**
	REV: TGGACTGGAAACCGTTTCAGA	
Osteopontin	FOR: CTGGCAGTGGTTTGCTTTTG	**AB001382**
	REV: CCACTTTCACCGGGAGACA	
Type 1 collagen	FOR: CGATGGCGTGCTATGCAA	**Z78279**
	REV: TCGCCCTCCCGTTTTTG	
Osterix	FOR: CATCTAACAGGAGGATTTTGGTTTG	**NM_053470**
	REV: AAGCCTTTGCCCACCTACTTTT	
Osteocalcin	FOR: AAGCCCAGCGACTCTGAGTCT	**NM_013414.1**
	REV: AGGTAGCGCCGGAGTCTATTC	
HIF-1α	FOR: GAACAAAACACACAGCGAAGCT	**XM_006240197.2**
	REV: TGCAGTGCAATACCTTCCATGT	
VEGF	FOR: TGTGCGGGCTGCTGCAATGAT	**AY702972.1**
	REV: TGTGCTGGCTTTGGTGAGGTTTGA	
GAPDH	FOR: AATGAAGGGGTCATTGATGG	**NM_017008.4**
	REV: AAGGTGAAGGTCGGAGTCAA	

## References

[b1] Kim H. K., Stephenson N., Garces A., Aya-ay J. & Bian H. Effects of disruption of epiphyseal vasculature on the proximal femoral growth plate. J Bone Joint Surg Am 91, 1149–1158 (2009). 19411464 10.2106/JBJS.H.00654

[b2] Park B. H. *et al.* COMP-Angiopoietin-1 ameliorates surgery-induced ischemic necrosis of the femoral head in rats. Bone 44, 886–892 (2009). 19442615 10.1016/j.bone.2009.01.366

[b3] Lee J. H. *et al.* Role of leptin in Legg-Calve-Perthes disease. J Orthop Res 31, 1605–1610 (2013). 23832827 10.1002/jor.22415

[b4] Friedman J. M. & Halaas J. L. Leptin and the regulation of body weight in mammals. Nature 395, 763–770 (1998). 9796811 10.1038/27376

[b5] Park B. H. *et al.* Combined leptin actions on adipose tissue and hypothalamus are required to deplete adipocyte fat in lean rats: implications for obesity treatment. J Biol Chem 281, 40283–40291 (2006). 17038325 10.1074/jbc.M607545200

[b6] Banks W. A. & Farrell C. L. Impaired transport of leptin across the blood-brain barrier in obesity is acquired and reversible. Am J Physiol Endocrinol Metab 285, E10–15; 10.1152/ajpendo.00468.2002 (2003). 12618361

[b7] Halaas J. L. *et al.* Weight-reducing effects of the plasma protein encoded by the obese gene. Science 269, 543–546 (1995). 7624777 10.1126/science.7624777

[b8] Chen H. *et al.* Evidence that the diabetes gene encodes the leptin receptor: identification of a mutation in the leptin receptor gene in db/db mice. Cell 84, 491–495 (1996). 8608603 10.1016/s0092-8674(00)81294-5

[b9] Turner R. T. *et al.* Peripheral leptin regulates bone formation. J Bone Miner Res 28, 22–34 (2013). 22887758 10.1002/jbmr.1734 PMC3527690

[b10] Bartell S. M. *et al.* Central (ICV) leptin injection increases bone formation, bone mineral density, muscle mass, serum IGF-1, and the expression of osteogenic genes in leptin-deficient ob/ob mice. J Bone Miner Res 26, 1710–1720 (2011). 21520275 10.1002/jbmr.406

[b11] Kalra S. P. *et al.* Leptin increases osteoblast-specific osteocalcin release through a hypothalamic relay. Peptides 30, 967–973 (2009). 19428775 10.1016/j.peptides.2009.01.020 PMC2749976

[b12] Enriori P. J., Evans A. E., Sinnayah P. & Cowley M. A. Leptin resistance and obesity. Obesity (Silver Spring) 14 **Suppl 5** 254S–258S (2006). 17021377 10.1038/oby.2006.319

[b13] Moran O. & Phillip M. Leptin: obesity, diabetes and other peripheral effects--a review. Pediatr Diabetes 4, 101–109 (2003). 14655266 10.1034/j.1399-5448.2003.00017.x

[b14] Turner R. T. *et al.* Morbid obesity attenuates the skeletal abnormalities associated with leptin deficiency in mice. J Endocrinol 223, M1–15 (2014). 24990938 10.1530/JOE-14-0224 PMC4161659

[b15] Ducy P. *et al.* Leptin inhibits bone formation through a hypothalamic relay: a central control of bone mass. Cell 100, 197–207 (2000). 10660043 10.1016/s0092-8674(00)81558-5

[b16] Thomas T. *et al.* Leptin acts on human marrow stromal cells to enhance differentiation to osteoblasts and to inhibit differentiation to adipocytes. Endocrinology 140, 1630–1638 (1999). 10098497 10.1210/endo.140.4.6637

[b17] Gordeladze J. O., Drevon C. A., Syversen U. & Reseland J. E. Leptin stimulates human osteoblastic cell proliferation, de novo collagen synthesis, and mineralization: Impact on differentiation markers, apoptosis, and osteoclastic signaling. J Cell Biochem 85, 825–836 (2002). 11968022 10.1002/jcb.10156

[b18] Steppan C. M., Crawford D. T., Chidsey-Frink K. L., Ke H. & Swick A. G. Leptin is a potent stimulator of bone growth in ob/ob mice. Regul Pept 92, 73–78 (2000). 11024568 10.1016/s0167-0115(00)00152-x

[b19] Kamohara S., Burcelin R., Halaas J. L., Friedman J. M. & Charron M. J. Acute stimulation of glucose metabolism in mice by leptin treatment. Nature 389, 374–377 (1997). 9311777 10.1038/38717

[b20] Scrocchi L. A., Brown T. J. & Drucker D. J. Leptin sensitivity in nonobese glucagon-like peptide I receptor -/- mice. Diabetes 46, 2029–2034 (1997). 9392491 10.2337/diab.46.12.2029

[b21] Shi Z. Q., Nelson A., Whitcomb L., Wang J. & Cohen A. M. Intracerebroventricular administration of leptin markedly enhances insulin sensitivity and systemic glucose utilization in conscious rats. Metabolism 47, 1274–1280 (1998). 9781634 10.1016/s0026-0495(98)90336-5

[b22] Tannenbaum G. S., Gurd W. & Lapointe M. Leptin is a potent stimulator of spontaneous pulsatile growth hormone (GH) secretion and the GH response to GH-releasing hormone. Endocrinology 139, 3871–3875 (1998). 9724042 10.1210/endo.139.9.6206

[b23] Kulkarni R. N. *et al.* Leptin rapidly suppresses insulin release from insulinoma cells, rat and human islets and, in vivo, in mice. J Clin Invest 100, 2729–2736 (1997). 9389736 10.1172/JCI119818 PMC508476

[b24] Seufert J., Kieffer T. J. & Habener J. F. Leptin inhibits insulin gene transcription and reverses hyperinsulinemia in leptin-deficient ob/ob mice. Proc Natl Acad Sci U S A 96, 674–679 (1999). 9892692 10.1073/pnas.96.2.674 PMC15195

[b25] Shimomura I., Hammer R. E., Ikemoto S., Brown M. S. & Goldstein J. L. Leptin reverses insulin resistance and diabetes mellitus in mice with congenital lipodystrophy. Nature 401, 73–76 (1999). 10485707 10.1038/43448

[b26] Hamrick M. W. *et al.* Leptin treatment induces loss of bone marrow adipocytes and increases bone formation in leptin-deficient ob/ob mice. J Bone Miner Res 20, 994–1001 (2005). 15883640 10.1359/JBMR.050103

[b27] Winet H. The role of microvasculature in normal and perturbed bone healing as revealed by intravital microscopy. Bone 19, 39S–57S (1996). 8830997 10.1016/s8756-3282(96)00133-0

[b28] Sierra-Honigmann M. R. *et al.* Biological action of leptin as an angiogenic factor. Science 281, 1683–1686 (1998). 9733517 10.1126/science.281.5383.1683

[b29] Cao R., Brakenhielm E., Wahlestedt C., Thyberg J. & Cao Y. Leptin induces vascular permeability and synergistically stimulates angiogenesis with FGF-2 and VEGF. Proc Natl Acad Sci U S A 98, 6390–6395 (2001). 11344271 10.1073/pnas.101564798 PMC33478

[b30] Ribatti D. *et al.* Angiogenic activity of leptin in the chick embryo chorioallantoic membrane is in part mediated by endogenous fibroblast growth factor-2. Int J Mol Med 8, 265–268 (2001). 11494053 10.3892/ijmm.8.3.265

[b31] Park H. Y. *et al.* Potential role of leptin in angiogenesis: leptin induces endothelial cell proliferation and expression of matrix metalloproteinases in vivo and in vitro. Exp Mol Med 33, 95–102 (2001). 11460888 10.1038/emm.2001.17

[b32] Kim H. K. *et al.* Increased VEGF expression in the epiphyseal cartilage after ischemic necrosis of the capital femoral epiphysis. J Bone Miner Res 19, 2041–2048 (2004). 15537448 10.1359/JBMR.040911

[b33] Ma H. Z., Zeng B. F. & Li X. L. Upregulation of VEGF in subchondral bone of necrotic femoral heads in rabbits with use of extracorporeal shock waves. Calcif Tissue Int 81, 124–131 (2007). 17629736 10.1007/s00223-007-9046-9

[b34] Garonna E. *et al.* Vascular endothelial growth factor receptor-2 couples cyclo-oxygenase-2 with pro-angiogenic actions of leptin on human endothelial cells. PLoS One 6, e18823; 10.1371/journal.pone.0018823 (2011). 21533119 PMC3078934

[b35] Ruiz M. *et al.* Activator protein 2alpha inhibits tumorigenicity and represses vascular endothelial growth factor transcription in prostate cancer cells. Cancer Res 64, 631–638 (2004). 14744778 10.1158/0008-5472.can-03-2751

[b36] Herz J. *et al.* Adenovirus-mediated transfer of low density lipoprotein receptor gene acutely accelerates cholesterol clearance in normal mice. Proc Natl Acad Sci U S A 90, 2812–2816 (1993). 8464893 10.1073/pnas.90.7.2812 PMC46186

