# Supplementary material for: Leptin ameliorates ischemic necrosis of the femoral head in rats with obesity induced by a high-fat diet
Source: Sci Rep. 2015 Mar 23;5:9397. doi: 10.1038/srep09397 (PMC4369691; doi:10.1038/srep09397)
Supplement: Supplementary Information [file srep09397-s1.pdf]

Leptin ameliorates ischemic necrosis of the femoral head in rats with obesity induced by a high-fat diet

Lu Zhou[1,4], Kyu Yun Jang[2], Young Jae Moon[3], Sajeev Wagle[1], Kyoung Min Kim[2]

Kwang Bok Lee[1], Byung-Hyun Park[3], Jung Ryul Kim[1,*]

[1]Department of Orthopaedic Surgery, Chonbuk National University Medical School, Research Institute for Endocrine Sciences and Research Institute of Clinical Medicine, Jeonju, Republic of Korea

[2]Department of Pathology, Chonbuk National University Medical School, Research Institute for Endocrine Sciences and Research Institute of Clinical Medicine, Jeonju, Republic of Korea

[3]Department of Biochemistry, Chonbuk National University Medical School, Research Institute for Endocrine Sciences, Jeonju, Republic of Korea

[4]Department of Sports Medicine, Taishan Medical University, Shandong, China

*Corresponding author:

Jung Ryul Kim, MD, PhD

Department of Orthopaedic Surgery, Chonbuk National University Medical School,

567 Baekje-daero, Deokjin-gu, Jeonju, 561-756, Republic of Korea.

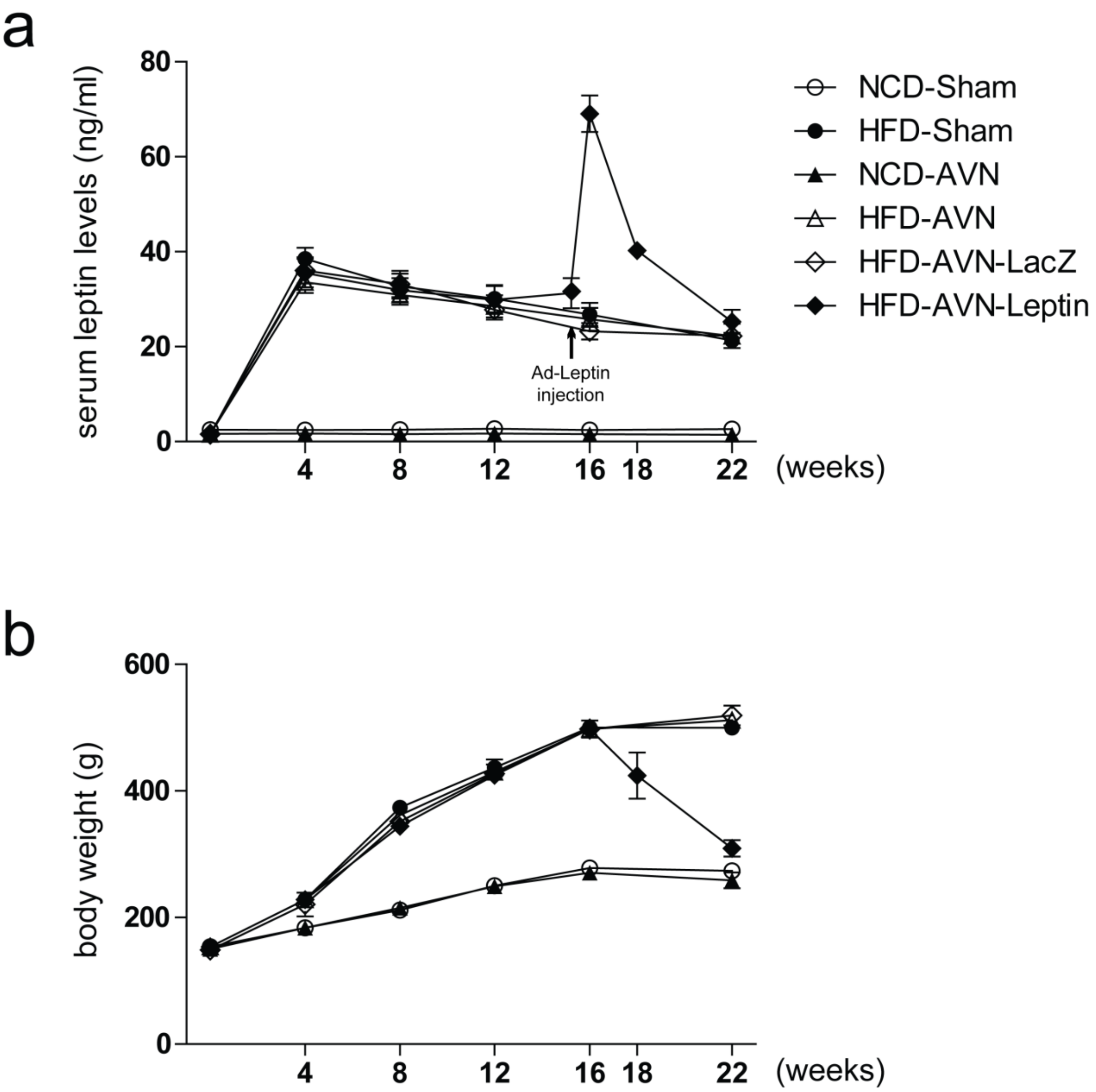

Supplementary Figure 1. Plasma leptin and body weight. Plasma leptin levels (A) and body weight (B) were measured in each group. Values represent mean ± SEM for four animals in each group.

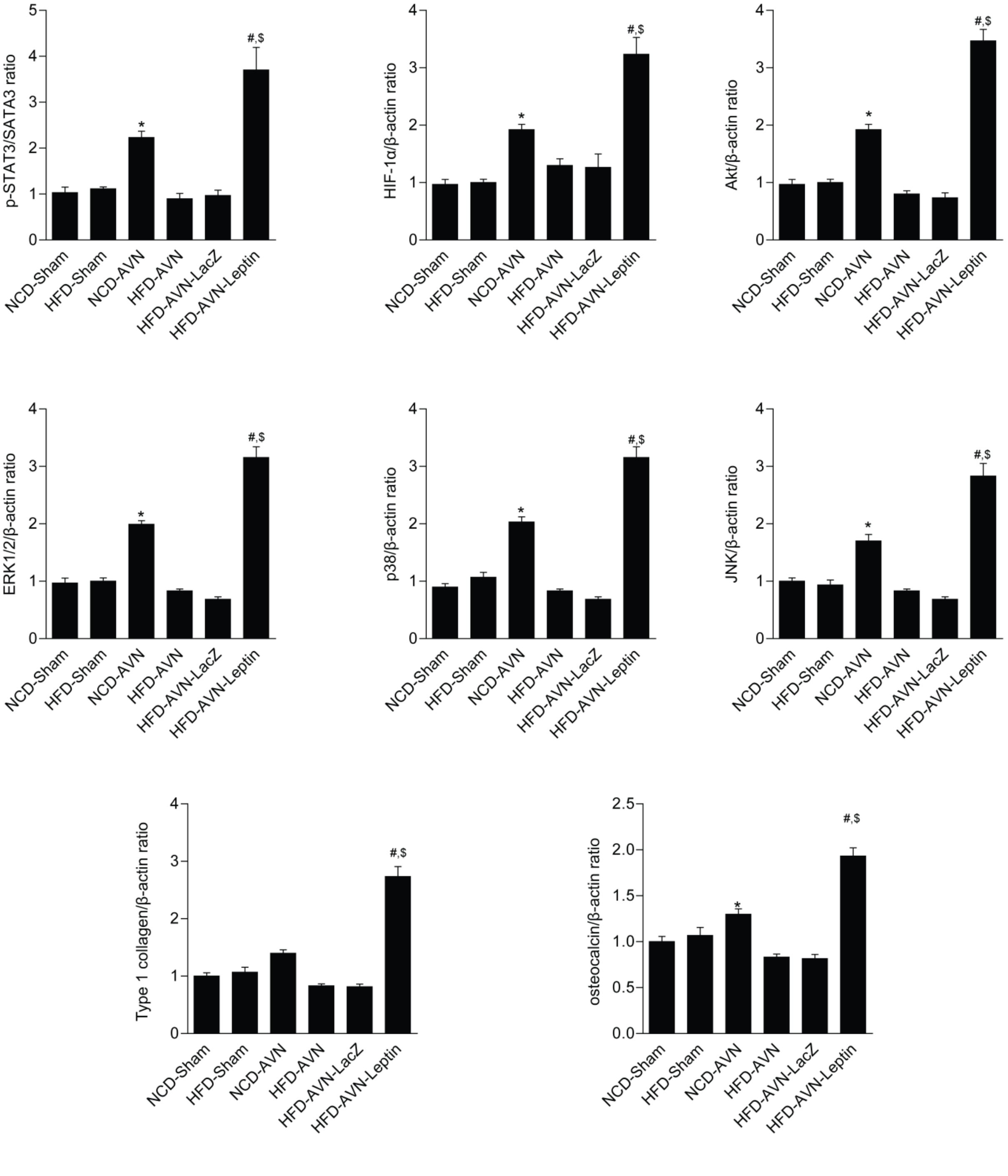

Supplementary Figure 2. Western blot analysis indicates both canonic and noncanonical leptin signaling pathways in the groups, respectively. The protein content was calculated by gray scale. Data are expressed as the mean SEM (n = 5 per group). *, $p < 0.05$ vs. NCD-Sham; $^{\#}$, $p < 0.05$ vs. NCD-AVN; $^{\$}$, $p < 0.01$ vs. NCD-AVN; $^{\&}$, $p < 0.01$ vs. HFD-AVN-LacZ

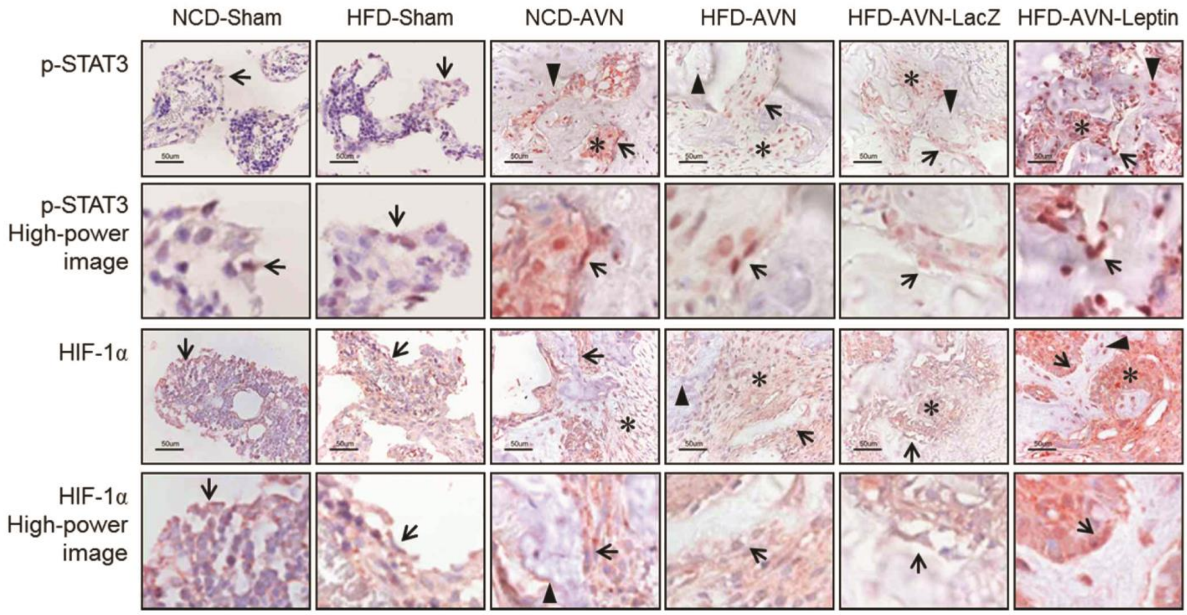

Supplementary Figure 3. High power images (H &E, x400) stromal cells on bone surfaces. Expression of phosphorylated STAT3 and HIF-1α were stronger in osteoblasts (arrows), osteoblasts (arrow heads), and stromal fibroblasts (asterisks) in HFD-AVN-Leptin compared with HFD-AVN or HFD-AVN-LacZ. Phosphorylated STAT3 and HIF-1α expression in NCD-AVN was intermediate between HFD-AVN-Leptin and HFD-AVN.

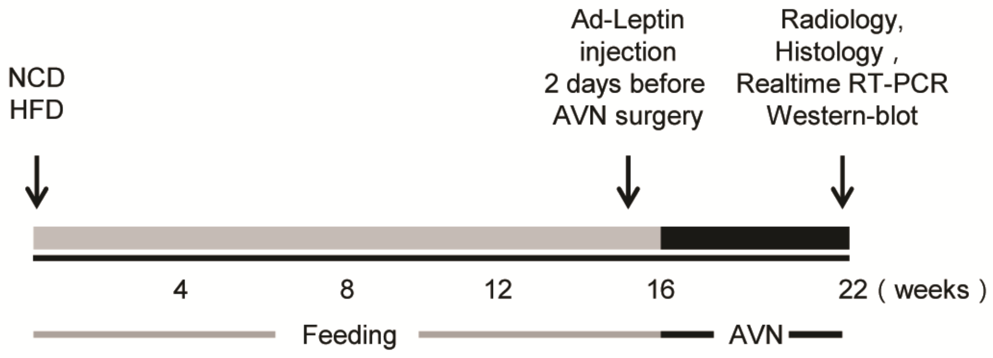

Supplementary Figure 4. Experimental design. After 16 weeks of NCD or HFD feeding, AVN was surgically induced. Ad-Leptin was by intravenous injection 2 days before surgery-induced AVN. Animals were euthanized at 6 weeks after inducing AVN.
